# Oxytocin and Vasopressin Agonists and Antagonists as Research Tools and Potential Therapeutics

**DOI:** 10.1111/j.1365-2826.2012.02303.x

**Published:** 2012-04

**Authors:** M Manning, A Misicka, A Olma, K Bankowski, S Stoev, B Chini, T Durroux, B Mouillac, M Corbani, G Guillon

**Affiliations:** * Biochemistry and Cancer Biology, University of Toledo College of MedicineToledo, OH, USA; † Faculty of Chemistry, University of WarsawWarsaw, Poland; ‡ Faculty of Chemistry, Institute of Organic Chemistry, Technical University of LodzLodz, Poland; § Pharmaceutical Research InstituteWarsaw, Poland; ¶ CNR Institute of NeuroscienceMilan, Italy; ** Institut de Genomique Fonctionnelle, UMR5203-CNRS, U661-INSERM, Univ. Montpellier I & IIMontpellier, Cedex, France

**Keywords:** vasopressin, oxytocin, agonists, antagonists, peptide, non-peptide

## Abstract

We recently reviewed the status of peptide and nonpeptide agonists and antagonists for the V_1a_, V_1b_ and V_2_ receptors for arginine vasopressin (AVP) and the oxytocin receptor for oxytocin (OT). In the present review, we update the status of peptides and nonpeptides as: (i) research tools and (ii) therapeutic agents. We also present our recent findings on the design of fluorescent ligands for V_1b_ receptor localisation and for OT receptor dimerisation. We note the exciting discoveries regarding two novel naturally occurring analogues of OT. Recent reports of a selective VP V_1a_ agonist and a selective OT agonist point to the continued therapeutic potential of peptides in this field. To date, only two nonpeptides, the V_2_/V_1a_ antagonist, conivaptan and the V_2_ antagonist tolvaptan have received Food and Drug Administration approval for clinical use. The development of nonpeptide AVP V_1a_, V_1b_ and V_2_ antagonists and OT agonists and antagonists has recently been abandoned by Merck, Sanofi and Pfizer. A promising OT antagonist, Retosiban, developed at Glaxo SmithKline is currently in a Phase II clinical trial for the prevention of premature labour. A number of the nonpeptide ligands that were not successful in clinical trials are proving to be valuable as research tools. Peptide agonists and antagonists continue to be very widely used as research tools in this field. In this regard, we present receptor data on some of the most widely used peptide and nonpeptide ligands, as a guide for their use, especially with regard to receptor selectivity and species differences.

## Introduction

Subsequent to the pioneering original synthesis of oxytocin (OT) (2) and arginine vasopressin (AVP) (3) by Vincent du Vigneaud and his associates, thousands of analogues of both of these neurohypophysial peptides have been synthesised in many laboratories throughout the world. The Merrifield solid phase method (4) has been of inestimable importance in facilitating the rapid and efficient synthesis of agonists and antagonists for the AVP V_1a_, V_1b_ and V_2_ receptors and for the OT uterine receptor (5). Many of these ligands have found widespread use as pharmacological tools for studies on the peripheral and central effects of OT and AVP. These design and synthetic studies have been the subject of numerous reviews (1, 6–20).

Structure activity and design studies carried out in other laboratories over the past five decades have laid the foundation for the design studies that we present here. These pivotal contributions by others have been fully documented (11, 14, 18, 20).

Oxytocin and AVP mediate their biological effects by acting on specific receptors (21–23). OT and AVP receptors belong to a G-protein coupled receptor family, characterised by seven putative transmembrane helices. Reviews on AVP and OT receptors are available elsewhere (12, 22, 23). OT receptors are expressed in the uterus, the mammary gland, the ovary, the brain, the kidney, the heart, bone and in endothelial cells (23). In the uterus, OT receptors mediate the uterine contracting (oxytocic) effect of OT (23). The central effects of OT continue to be the focus of intense investigative scrutiny in animals (24–28) and in humans (29–35), as a possible therapeutic agent for the treatment of autism and other anxiety disorders.

Arginine vasopressin mediates its actions through three known receptors: V_1a_, V_1b_ and V_2_. V_1a_ receptors are expressed in the liver, vascular smooth muscle cells, brain and in many other tissues (12, 21, 22). In the vasculature, V_1a_ receptors mediate the pressor actions of AVP by a phospholipase C-mediated pathway. In the brain, V_1a_ receptors mediate the anxiety producing responses to AVP (27, 36). V_1b_ receptors, discovered long after the V_1a_ and V_2_ receptors, present in the anterior pituitary, mediate the adrenocorticotrophic hormone-releasing effects of AVP, also by a phospholipase C-mediated pathway (22) Evidence for the presence of V_1b_ receptors in extra-pituitary tissues such as brain, the kidney and the adrenal medulla has also been reported (37). Recently, the V_1b_ receptor has been shown to mediate anxiety and stress in rats and in humans (45). V_2_ receptors, present in the collecting duct of the kidney, mediate the antidiuretic action of AVP by an adenylate cyclase-mediated pathway (12, 21, 22).

## Scope of the present review

We have previously reviewed the status of developments in the design and synthesis of peptide and nonpeptide AVP and OT agonists and antagonists (1). Here, we focus on the properties of the most widely used peptides requested from the Manning laboratory or purchased from suppliers, together with some recently reported potential clinically useful peptides from the Ferring Laboratory (38, 39). Space considerations preclude our being able to present or to discuss recent synthetic studies carried out in other laboratories (40–43). We also update the current status of the pre-clinical and clinical development of nonpeptide AVP and OT antagonists and of the pre-clinical development of nonpeptide OT agonists (44). The excellent reviews on nonpeptide AVP antagonists (45) and on nonpeptide OT antagonists (46) should be consulted for more in-depth presentations of their chemistry and pharmacology. We also review the merits of peptide versus nonpeptide AVP and OT agonists and antagonists as: (i) research tools and (ii) therapeutic agents. We present human and rat receptor data for a number of selective peptide agonists and for both peptide and nonpeptide antagonists. We illustrate the need to be aware of: (i) species differences, (ii) selectivity differences and (iii) *in vitro–in vivo* differences when using a specific ligand for receptor characterisation. Finally, we present the highlights of our recent studies aimed at: (i) the development of selective fluorescent ligands for the rat and human V_1b_ receptors (47) and (ii) the development of fluorescence based strategies that have been used to prove the existence of OT receptor dimers in native tissue (48).

## Peptide synthesis

All the OT and AVP agonists, antagonists, radiolabelled and fluorescent ligands from our laboratories were synthesised using the Merrifield solid-phase method (4, 49). The synthetic strategy relies very heavily on methodology developed in the du Vigneaud laboratory for the original syntheses of OT and AVP (2, 3). The procedures used are described in the original publications cited here. For other references, see Manning *et al.* (1).

## Bioassays

All of the published peptides from our laboratories, presented in Tables 1, 3–8, were assayed for agonistic and antagonistic activities in *in vitro* and *in vivo* rat oxytocic assays, in the rat vasopressor assay and in the rat antidiuretic assay in the laboratories of our collaborators Drs Wilbur H. Sawyer, W. Y. Chan and Hazel Szeto. For agonists, the four-point assay design (50) was used and for antagonists, Schild’s pA_2_ method (51) was employed. The pA_2_ is the negative logarithm of the molar concentration of the antagonist that requires a two-fold increase in agonist concentration to achieve the same effect as that found in the absence of antagonist. In practice, this concentration is estimated by finding concentrations above and below the pA_2_ dose and interpolating on a logarithmic scale.

In the rat *in vivo* assays, the pA_2_ (effective dose) is divided by an arbitrarily assumed volume of distribution of 67 ml/kg (52) in an attempt to derive the approximate molar concentration [M] of the pA_2_ dose in the vicinity of the receptors. Thus, *in vivo* pA_2_ values are very approximate estimates. The USP Posterior Pituitary Reference Standard or synthetic OT and AVP, which had been standardised in oxytocic and vasopressor units against this standard, were used as agonists for working standards in all bioassays. *In vitro* oxytocic assays were performed on isolated uteri from diethylstilbestrol-primed rats in a Mg^2+^-free van Dyke Hasting’s solution (53). *In vivo* anti-OT potencies were determined in urethane-anaesthetised diethylstilbestrol-primed rats as described previously (54, 55). Vasopressor assays were performed on urethane-anaesthetised and phenoxybenzamine-treated rats as described by Dekanski (55), and antidiuretic assays on water-loaded rats under ethanol anesthesia as described by Sawyer (56).

## Receptor binding and functional assays

Membranes and/or cell lines that express the rat and human AVP V_1a_, V_1b_ and V_2_ receptors (57–64) and the human OT receptor (65) were used for binding and functional assays: inositol phosphate accumulation (66) for V_1a_, V_1b_ and OT receptors and cyclic AMP accumulation (67) for V_2_ receptors, as described previously (68–74). These receptor studies were carried out in Montpellier and Milan.

## Selective oxytocin agonists (Table 1)

**Table 1:** Potent and Selective Agonists for the Uterine Oxytocin Receptor in the Rat.

					Ratios		
Number	Peptide	OT receptor oxytocic (O) (units/mg)	V_1a_ receptor vasopressor (P) (units/mg)	V_2_ receptor antidiuretic (A) (units/mg)	O/A	O/P	Reference
	OT	520	4	4	130	130	75
1	[Thr^4^]OT	923	0.4	0.9	1025	2307	75
2	HO[Thr^4^]OT	4179	4.92	5.3	790	850	75
3	[Thr^4^, Gly^7^]OT	166	< 0.01	∼0.002	83 000	>16 600	75
4	HO[Thr^4^, Gly^7^]OT	218	< 0.01	0.004	54 500	>21 800	75
5	Carba-1-[4-FBzlGly^7^]dOT(FE 202767)^*^	ND	ND	ND			38

OT, oxytocin; HO, 1-hydroxy (hydroxyl group replaces α-amino group); FBzl, fluorobenzyl. ^*^In vitro potency EC_50_^a^ (nm) hOT 0.08, hV_2_ 330, hV_1a_>10000 selectivity versus receptor hV_2_ 4100, hV_1b_ > 120000. ^a^EC_50_ is the concentration of agonist leading to half-maximal activity. ND, Not determined.

The pharmacological properties in rat bioassays for OT and the four analogues (peptides **1**–**4**), which are more potent and/or more selective than OT (75), are given in Table 1. [Thr^4^,Gly^7^]OT (peptide **3**), also referred to as TGOT, has been widely used as a selective OT agonist. Its human and rat receptor affinities are given in Table 11. The recently reported (38) highly selective OT analogue FE 202767 (peptide **5**) has not been evaluated in standard rat bioassays. It exhibits high affinity and selectivity for the human OT receptor. It thus offers promise as a potential new OT therapeutic (38).

The two new OT-related analogues given in Table 2 (1, 2) have not yet been evaluated in standard rat bioassays. The discovery of [Pro^8^]OT in new world monkeys by Lee *et al*. (76), independently confirmed by Jeffrey French and colleague (E. B. Harrison and J. A. French, personal communication), is an exciting new development in this field. The novel C-terminal extended analogue of OT; oxytocin-Gly-Lys-Arg, reported by Gutkowska *et al*. (77) and Danalache *et al*. (78), opens up new possibilities for the design of potential new cardiomyogenic therapies.

**Table 2:** New Oxytocin (OT)-Related Peptides.

Number	Peptide	Reference
1	Oxytocin-Gly-Lys-Arg	77, 78
2	[Pro^8^]OT	76

## Selective vasopressin V_2_ receptor agonists (Table 3)

**Table 3:** Potent and Selective Agonists for the Vasopressin V_2_ Receptor in the Rat^a^.

					Ratios		
Number	Peptide	OT receptor oxytocic (O) (units/mg)	V_1a_ receptor vasopressor (P) (units/mg)	V_2_ receptor antidiuretic (A) (units/mg)	A/P	A/O	Reference
	AVP	14	373	320	0.9	22.8	79
1	dDAVP (desmopressin)^*^	1.5	0.39	1200	3000	800	79, 80
2	VDAVP	0.60	0.037	653	17 650	1 090	79
3	dVDAVP	8	Antagonist (pA_2_ = 7.03)	1230	Infinite		79

OT, oxytocin; AVP, arginine vasopressin; d, 1-deamino; DAVP, D-Arg^8^VP; V, Val^4^. ^*^Desmopressin is the drug of choice for the treatment of diabetes insipidus. ^a^The data given are obtained from Sawyer *et al.* (79).

AVP is equipotent as an antidiuretic agonist and as a vasopressor agonist (79) (Table 3). Thus, it is totally nonselective. It is also not selective with respect to its oxytocic activity. The three analogues of AVP, peptides **1**–**3** in Table 3 namely; dDAVP, VDAVP and dVDAVP, exhibit striking gains in antidiuretic/vasopressor selectivity. All three peptides have been widely used as selective V_2_ agonists. dDAVP, first synthesised by the Zaoral *et al*. (80) in Prague and later licensed to Ferring, has long been the drug of choice for the treatment of diabetes insipidus. It has been marketed under the trademark Desmopressin (Minirin). The human receptor affinities for dDAVP and dVDAVP given in Table 11 shows clearly that dVDAVP has a ten-fold higher affinity for the human VP V_2_ receptor than dDAVP. However, both peptides also exhibit high affinities for the human V_1b_ receptor and to somewhat lesser extent for the human OT receptor (Table 11). So clearly they are not selective V_2_ agonists in humans with respect to both hV_1b_ or hV_1a_ receptors. The search for a V_2_ agonist that is selective with respect to the V_1a_ and V_1b_ receptors in humans is still a challenging goal in this field. Yet, in the rat, dDAVP could be considered as a relatively good selective V_2_ agonist (Table 11).

## Selective vasopressin V_1a_ receptor agonists (Table 4)

**Table 4:** Potent and Selective Agonists for the Vasopressin V_1a_ Receptor in the Rat.

					Ratios		
Number	Peptide	OT receptor oxytocic (O) (units/mg)	V_1a_ receptor vasopressor (P) (units/mg)	V_2_ receptor antidiuretic (A) (units/mg)	P/A	P/O	Reference
	AVP	373	320	14	1.2	26.6	79, 84
	LVP ([Lys^8^]VP)	270	284	10	0.95	27	85
1	[Phe^2^]LVP (felypressin, octapressin)	57	21	0.3	2.7	190	17
2	[Phe^2^]OVT, [Phe^2^,Orn^8^]vasotocin	124	0.55	1	225	124	81
3	F-180	164	0.19		863		82
4	FE 202158^*^	ND	ND	ND			39, 83

OT, oxytocin; F180, Hmp-Phe-Ile-Hgn-Asn-Cys-Pro-Dab(Abu)-Gly-NH_2_; where Hmp, 2-hydroxy-3-mercaptopropionic acid; Hgn, homoglutamine; Dab, 2,4-diaminobutyric acid; Abu, 2-aminobutyric acid; ^*^FE 202158, [Phe^2^,Ile^3^,Hgn^4^,Orn(iPr)^8^]AVP, where Hgn is homoglutamine and iPr is isopropyl.

In rat bioassays, [Phe^2^]OVT (peptide **2**; Table 4) is a fairly potent vasopressor agonist (81). Its vasopressor (P) activity is 124 units/mg. In antidiuretic (A) assays, it exhibits only 0.55 units/mg. Its P/A ratio is 225 (81). Thus, for many years, it has been considered to be a selective V_1a_ agonist and has been widely used as a selective V_1a_ agonist. However, based on its rat V_1a_ receptor affinity data in Table 11, it is not selective for the rat V_1a_ receptor in this assay. In this regard, the selective V_1a_ agonist F-180 (82), which is a highly selective V_1a_ agonist in rat bioassays (peptide **3**; Table 4), is even more puzzling. In rat receptor assays (Table 11), it is clearly nonselective for V_1a_ receptors. By contrast, F-180 exhibits high affinity and selectivity for the human V_1a_ receptor. The exciting new V_1a_ agonist (peptide **4**; Table 5), FE202158, recently reported by Ferring (39, 83) is currently undergoing clinical trials for the treatment of vasodilating hypotension. Compared to F180, this peptide exhibits better selectivity for the human V_1a_ receptor (Table 11). In the rat, this agonist is very specific for the V_1a_ receptor compared to the V_2_ receptor (selectivity higher than 800), yet it has not been tested for the OT and V_1b_ receptors. This intriguing new V_1a_ agonist is not yet available to other scientists for use as a pharmacological research tool.

**Table 5:** Lys^8^ Analogues of d[Cha^4^]AVP (1) and d[Leu^4^]AVP (3) exhibit High Affinities and Selectivities for both Rat and Human V_1b_ Receptors.

			Affinity (K_i_) (nm)								
Number	Peptide	Rat antidiuretic activity (U/mg)	rV_1b_-R	hV_1b_-R	rV_2_-R	hV_2_-R	rV_1a_-R	hV_1a_-R	rOT-R	hOT-R	Reference
	AVP	323	0.29	0.68	0.45	1.2	2.6	1.1	1.7	1.7	74, 84
	dAVP	1745	0.20	0.37	0.76	5	10.8	3.8	0.97	–	74, 84
1	d[Cha^4^]AVP	133.6	1.40	1.2	12.7	750	2297	151	1430	240	69, 72, 74
2	d[Cha^4^, Lys^8^]VP	0.82	1.9	2.2	596	11 484	9093	283	586	141	85, 86
3	d[Leu^4^]AVP	378	0.04	0.23	3.1	245	1252	44.1	481	211	72, 74
4	d[Leu^4^, Lys^8^]VP	10.5	0.16	0.51	101	6713	3786	69.3	64	29	74, 86, 87

## V_1b_ receptor agonists (Table 5)

AVP was synthesised in 1954 (3) (Table 5).The first ‘selective’ V_1a_ agonist [Phe^2^]OVT was synthesised 10 years later in 1964 (81). The first selective V_2_ agonist dDAVP was reported in 1967 (80). Yet, it was not until 2002, almost 50 years after the synthesis of AVP, that the first selective agonist for the human V_1b_ receptor, d[Cha^4^]AVP was synthesised (69). The reasons why this discovery took so long have been documented by Manning *et al.* (1).

Table 5 lists four analogues of dAVP (peptides **1**–**4**) that exhibit high affinities for both the rat and human V_1b_ receptors. d[Cha^4^]AVP (peptide **1**) was the first V_1b_ agonist that was shown to be selective for the human V_1b_ receptor (69). d[Leu^4^]AVP (peptide **3**) has later been shown to be a selective agonist for the human V_1b_ receptor (72). Both (peptides **1** and **3**) exhibit high affinities for the rat V_1b_ receptor. However, they also possess high *in vivo* antidiuretic activity. Thus, neither is a selective V_1b_ agonist in the rat.

Replacement of the Arg^8^ residue in (peptides **1** and **3**) by a Lys^8^ residue to give d[Cha^4^,Lys^8^]VP (peptide **2**) and d[Leu^4^,Lys^8^]VP (peptide **4**), respectively, resulted in the first peptides that are selective V_1b_ agonists in the rat (73, 74). It was subsequently shown that both peptides **1** and **2** are also highly selective for human V_1b_ receptors (86). It bears noting that d[Leu^4^,Lys^8^]VP had been reported to be a weak antidiuretic V_2_ agonist/weak vasopressor agonist in the rat (87), 30 years before the V_1b_ receptor was first predicted and/or cloned (88). The selective V_1b_ agonists d[Cha^4^]AVP and d[Leu^4^,Lys^8^]VP have been used as a research tool in a number of studies (1, 20, 62, 73, 89, 90). Furthermore, d[Leu^4^,Lys^8^]VP has been utilised in the design of a series of fluorescent ligands for the V_1b_ receptor (47).

## Selective V_1a_ antagonists (Table 6)

**Table 6:** Design of Highly Selective V_1a_ Antagonists.

		Anti-OT (*in vitro*)	Anti-OT (*in vivo*)	Anti-V_1a_ (*in vivo*)		
Number	Peptide	pA_2_^a^	pA_2_^b^	pA_2_^b^	Anti-V_1a_/anti-OT selectivity	Reference
1	d(CH_2_)_5_[Tyr(Me)^2^]AVP (Manning compound)	8.13	6.62	8.62	100	91
2	d(CH_2_)_5_[Tyr(Me)^2^, Dap^5^]AVP	5.83	ND^c^	7.49	Infinite	92
3	d(CH_2_)_5_[Tyr(Me)^2^, Dab^5^]AVP	ND^c^	ND^c^	6.71	Infinite	92

OT, oxytocin; d(CH)_5_ = β-mercapto-β,β-cyclopentamethylenepropionyl. ^a^*In vitro* pA_2_ values represent the negative logarithm to the base 10 of the average molar concentration [m] of the antagonist that reduces the response to 2 × units of agonist to the equal the response seen with 1 × units of agonist administered in the absence of the antagonist. ^b^*In vivo* pA_2_ values are estimated because the molar concentration for the in vivo pA_2_ is estimated by dividing the effective dose (ED) by the estimated volume of distribution of (67 ml/kg) (52). ED is defined as the dose (nmol/kg intravenously) of the antagonist that reduces the response to 2 × units of agonist to the response with 1 × units of agonist administered in the absence of the antagonist. ^c^ND, not detectable (weak agonist, < 0.03 U/mg).

d(CH_2_)_5_[Tyr(Me)^2^]AVP, also known as Manning compound (peptide **1**; Table 6) is a potent VP V_1a_ antagonist/weak VP V_2_ agonist (91). It is thus highly selective for V_1a_ receptors versus V_2_ receptors. It is, however, a potent *in vitro* OT antagonist and a fairly potent OT antagonist *in vivo* (91). It has found widespread use as a selective V_1a_ antagonist in a variety of studies on the peripheral and central effects of AVP. Indeed, it has become the most widely used V_1a_ antagonist reported to date. Isosteric modifications of d(CH_2_)_5_[Tyr(Me)^2^]AVP at position 5 with diaminopropionic acid (Dap) and diaminobutyric acid (Dab) led to d(CH_2_)_5_[Tyr(Me)^2^,D_ap_^5^]AVP (peptide **2**; Table 6) and d(CH_2_)_5_[Tyr(Me)^2^,Dab^5^]AVP (peptide **3**; Table 6), respectively. Both peptides are devoid of anti OT activity *in vivo* (92). Although both peptides are much less potent than d(CH_2_)_5_[Tyr(Me)^2^]AVP as V_1a_ antagonists, because they lack anti OT potency *in vivo,* they are highly selective for V_1a_ receptors in the rat. Their use is recommended for *in vivo* studies that require discrimination between V_1a_ and OT receptors in the rat.

## Nonselective and selective cyclic and linear V_2_/V_1a_ antagonists for rat receptors

It was not until 1981, almost 30 years after the first laboratory synthesis of OT, that the first cyclic AVP V_2_/V_1a_ antagonists were reported (8) (Table 7). Six years later, the unexpected discovery of the first linear V_2_/V_1a_ antagonists was reported (93). The early cyclic and linear V_2_/V_1a_ antagonists were nonselective for V_2_ receptors. Further modifications of the early cyclic V_2_/V_1a_ antagonists led to the discovery of selective cyclic V_2_ antagonists (94). Some of the most commonly used nonselective cyclic and linear V_2_/V_1_ antagonists (peptides **1**, **2**, **7**) and selective cyclic V_2_ antagonists (peptides **3**–**6**) are given in Table 7. Peptide **6** (101) has been very useful for the design of a lanthanide cryplate-labelled ligand as a fluorescent probe for measuring receptor dimerisation (48) Peptide **8** (HO-LVA) (95), a potent linear V_1a_ antagonist, has served as a precursor for the radioiodinated V_1a_ ligand [^125^I]HO-LVA (97). This radioligand has found widespread use as a selective probe for V_1a_ receptors. Its affinities for rat receptors are given in Table 12. A number of these V_2_/V_1a_ antagonists (peptides **1**–**3**; Table 7) exhibit oxytocic antagonism *in vivo*. The remaining peptides **4**–**8** have not been evaluated in anti-OT assays. Caution should be exercised in using any of the peptides in Table 7 as selective V_2_ ligands. The affinities of peptides **1** and **5** for the human and rat V_2_ receptors are given in Table 12.

**Table 7:** Nonselective and Selective Cyclic and Linear V_2_/V_1a_ Antagonists for Rat Receptors.

		Antiantidiuretic (A) (anti-V_2_)		Antivasopressor (P) (anti-V_1a_)		Antioxytocic *In vivo*		
Number	Peptide	ED^a^	pA_2_^b^	ED^a^	pA_2_^b^	pA_2_^b^	ED ratio A/P	Reference
1	d(CH_2_)_5_[D-Tyr(Et)^2^]VAVP	1.1	7.81		8.22	7.47^*^	0.4	98
2	desGly,d(CH_2_)_5_[D-Tyr(Et)^2^, Val^4^]AVP	1.5	7.69	0.45	8.17	6.98^*^	0.3	99
3	d(CH_2_)_5_[D-Ile^2^, Ile^4^]AVP	0.67	8.04	0.45	6.42	6.90^*^	39	94
4	desGly-NH_2_,d(CH_2_)_5_[D-Ile^2^, Ile^4^]AVP	0.90	7.88	26	∼5.2		∼440	100
5	d(CH_2_)_5_[D-Ile^2^, Ile^4^, Ala-NH_2_^9^ ]AVP	0.46	8.16	∼400	6.25		83	100
6	d(CH_2_)_5_[D-Tyr(Et)^2^, Ile^4^, Eda^9^]AVP	0.77	8.00	38	8.33			101
7	Aaa-D-Tyr(Et)-Phe-Val-Asn-Abu-Pro-Arg-Arg-NH_2_	0.53	8.11	0.32	7.75		2.3	48, 95
8	4-HO-Phaa-D-Tyr(Me)-Phe-Gln-Asn-Arg-Pro-Arg-NH_2_(HO-LVA)	Agonist	0.056 U/mg	1.2	8.47			12, 96, 97

Aaa, adamantaneacetyl; Eda, ethylenediamine; 4-HO-Phaa, 4-hydroxyphenylacetyl. ^*^In vivo anti-oxytocin (OT) potencies were reported previously (10), ^a^The effective dose (ED) is defined as the dose (in nmol/kg) that reduces the response to 2 × units of agonist to equal the response to 1 × unit. ^b^ Estimated *in vivo* pA_2_ values represent the negative logarithms of the EDs divided by the estimated volume of distribution (67ml/kg) (52).

## Some nonselective and selective oxytocin antagonists (Table 8)

**Table 8:** Some Nonselective and Selective Oxytocin Antagonists in the Rat.

		Antioxytocic (anti-OT)							
		*In vitro* pA2^a^	*In vivo*		Antivasopressor (anti-V_1a_)		Antidiuretic activity (V_2_)		
Number	Peptide	No Mg^2+^	ED^a^	pA_2_^c^	ED^b^	pA_2_^c^	Units/mg	ED ratio^d^	Ref
1	d[D-Tyr(Et)^2^,Thr^4^]OVT (atosiban)	8.29	Antagonist		Agonist 0.02 (IU/μmol)		Agonist 0.04 (IU/μmol)		106
		7.71	5.95	7.05	48.5	6.14	Antagonist (pA_2_ ≍ 5.9)	8	104
2	d(CH_2_)_5_[Tyr(Me)^2^]OVT	8.52	4.2	7.37	0.80	7.96	≍ 0.01	0.2	103
3	desGly-NH_2_,d(CH_2_)_5_[Tyr(Me)^2^, Thr^4^]OVT	7.89	1.3	7.69	23	6.48	Antagonist (pA_2_ ≍ 5.5)	17.7	104
4	d(CH_2_)_5_[Tyr(Me)^2^, Thr^4^, Tyr-NH_2_^9^ ]OVT	7.63	1.0	7.83	6.6	7.02	≍ 0.015	6.6	104, 105
5	desGly-NH_2_,d(CH_2_)_5_[D-Tyr^2^, Thr^4^]OVT	7.77	2.85	7.37	272	5.39	Antagonist (pA_2_ < 5.5)	95	104

a *In vitro* pA_2_ values represents the negative logarithm to the base 10 of the average molar concentration [m] of antagonist which reduces the response to 2 × units of agonist to the response with × units of agonist. ^b^The effective dose (ED) is defined as the dose (in nm/kg) of antagonist that reduces the response to 2 × units of agonist to the response with × units of agonist administered in the absence of antagonist. ^c^Estimated *in vivo* pA_2_ values represent the negative logarithms of the ‘effective dose’ divided by the estimated volume of distribution (67 ml/kg) (52). ^d^ED ratio = anti-vasopressor ED/antioxytocic ED.

The OT antagonists listed in Table 8 have all found widespread use as pharmacological tools. Under the tradename Tractocile, atosiban (peptide **1**) has been approved for clinical use in Europe for the prevention of premature labour (102). All of these OT antagonists exhibit varying degrees of anti-V_1a_ potency in the rat. Thus, they are far from being selective. Indeed, d(CH_2_)_5_[Tyr(Me)^2^]OVT, one of our original OT antagonists (103), is five-fold more potent as a vasopressor antagonist than as an OT antagonist (103). Peptide **5**, desGly-NH_2_, d(CH_2_)_5_ [D-Tyr^2^,Thr^4^]OVT (104) is the most selective OT antagonist in Table 8. It has been used in a variety of studies. These are listed under ‘Research Uses’.

This OT receptor antagonist also exhibits a high affinity and selectivity for the human OT receptor (Table 12). Peptide **3**, desGly-NH_2_,d(CH_2_)_5_[Tyr(Me)^2^Thr^4^]OVT, although much less selective than OTA No. 5, has proved to be very useful as a research tool in the Neumann laboratory for a wide variety of studies on the central effects of OT. Some examples of these studies are listed under ‘Research Uses’.The ^125^I derivative of peptide 4, d(CH_2_)_5_[Tyr(Me)^2^,Thr^4^,Tyr-NH_2_^9^]OVT (105) has found widespread use as a probe of OT receptors.

## Nonpeptide vasopressin antagonists as pharmacological tools and therapeutic agents (Table 9)

**Table 9:** Nonpeptide Vasopressin Antagonists as Pharmacological Tools and Therapeutic Agents.

Number	Receptor Type	Company	Code	Name	Supplier	Status	Reference: synthesis	Reference: pharmacological use	Reference: clinical use
1	V_1a_	Sanofi	SR49059	Relcovaptan	Tocris	Phase II (terminated)	45, 116	117	
2	V_1a_	Pfizer	PF-00738245	No name	Pfizer	New compound	118		
3	V_1a_	Otsuka	OPC-21268	No name	Tocris Sigma-Aldrich	Phase II Japan stopped US/Europe	107		
4	V_1b_	Sanofi	SSR149415	Nelivaptan	Axon Medchem	Preclinical (terminated)	45, 119, 164	120, 147, 148, 150	
5	V_2_	Otsuka	OPC-41061	Tolvaptan	Shanghai DND Pharm-Technology Co.,Inc.	Approved by US Food and Drug Administration oral use (Samsca)	108	115	111–115, 121–123
6	V_2_	Sanofi	SR121463(B)	Satavaptan	None	Phase III (terminated)	45	124–126	
7	V_2_	Otsuka	OPC-31260	Mozavaptan	Otsuka; Anhui Pharmaceutical Co., LTD	Phase II	127		
8	V_2_/V_1a_	Astellas	YM-087	Conivaptan	LGM Pharma, Beijing HuameiHuli Biochem Ltd	Approved by US Food and Drug Administration i.v. use (Vaprisol)	127	152	111, 112, 128, 129

The search for nonpeptide antagonists for the AVP, V_1a_, V_1b_ and V_2_ receptors has been pursued with vigour by many pharmaceutical companies, most notably Otsuka, Sanofi, Azevan, Astellas, Wyeth-Ayerst, Johnson & Johnson, Yamanouchi and Pfizer (Table 9). In 1991, Otsuka reported the first nonpeptide V_1a_ antagonist OPC-21268 (No. **3**; Table 9) (107). During the subsequent 20 years, a number of promising nonpeptide V_1_, V_1b_, and V_2_ antagonists have been reported (1, 45). Table 9 lists a number of these, together with references to: (i) their original synthesis (45, 107, 108, 110, 116–119, 127, 164); (ii) some research uses (115, 117, 120, 124, 126, 147, 148, 150, 152); and (iii) their clinical uses (109, 111–115, 121-123, 128, 129). Most notable, are the Otsuka V_2_ antagonist (Tolvaptan, No. **5**; Table 9) first reported in 1998 by Yamamura *et al.*, (108) and shown to be effective in the treatment of hyponatraemia (109) and the Astellas V_2_/V_1a_ antagonist (Conivaptan, No. **8**; Table 9) first reported in 1997 by Tahara *et al.* (110). Both conivaptan and tolvaptan have been approved for clinical use by the Food and Drug Administration (111–115). The development of nonpeptide V_1a_, V_1b_ and V_2_ antagonists at other companies has recently been abandoned as a resut of failures in clinic trials. Thus, Sanofi, which had reported the V_1a_ antagonist SR-49059, Relcovaptan (No. **1**; Table 9), the V_1b_ antagonist SSR-149415, Nelivaptan (No. **4**; Table 9) and the V_2_ antagonist SR-121463 (B) Satavaptan (No. **6**; Table 9), all highly promising candidates for therapeutic development (45), has recently abandoned its entire AVP nonpeptide antagonist programme. It should be noted that these three Sanofi nonpeptide antagonists are highly useful pharmacological tools. The commercial availability of some of these nonpeptide VP antagonists is shown in Table 9. Human and rat receptor affinities for all three Sanofi V_1a_, V_1b_ and V_2_ nonpeptides are given in Table 12.

## Nonpeptide oxytocin antagonists and a nonpeptide oxytocin agonist (Table 10)

**Table 10:** Non peptide Oxytocin Antagonists and a Nonpeptide Agonist.

Compound	Number	Code (name)	Company	Supplier	Status	Reference: original synthesis, structure and pharmacologocal properties)	Reference: use
OT antagonist	1	L-368,899	Merck	Tocris	Phase II Discontinued	46, 130	134–136
	2	L-371,257	Merck	Tocris	Phase II Discontinued	46	141
	3	WAY-162720	Wyeth-Ayerst (now Pfizer)	No	Failed in preclinical	131	141
	4	GSK2211149A (Retosiban)	Glaxo SmithKline	Simagchem; Manus Aktteva Biopharma LLP	Phase II completed	132, 133	
OT agonist	1	WAY-267464	Wyeth-Ayerst (now Pfizer)	Tocris	Failed in preclinical	138, 140	137, 141

A number of companies have been active in this area. In the mid-1990s, Merck reported a number of promising nonpeptide OT antagonists (46, 130) (Table 10). Most notable were L-368,899 (No. **1**; Table 10) and L-371, 257 (No. **2**; Table 10). Both of these failed in clinical development for the treatment of premature labour. Merck subsequently abandoned its nonpeptide OT antagonist programme. Similarly, Wyeth-Ayerst (now Pfizer) reported that its nonpeptide OT antagonist WAY-1627720 (131) (No. **3**; Table 10) failed in preclinical development. Glaxo SmithKline is now the only company pursuing the clinical development of a nonpeptide OT antagonist. Its promising nonpeptide OT antagonist GSK 2211149A (Retosiban) (132, 133) is currently in a Phase II clinical trial. Its human and rat OT receptor affinities are given in Table 12. Its OT selectivities for human OT/VP receptors are excellent. Although the Merck and Wyeth-Ayerst nonpeptide OT antagonists have failed in clinical development, they are proving to be very useful as research tools (134–138) The Merck compounds are now available from TOCRIS. The nonpeptide OT agonist WAY-267464 reported by Pfizer (131) appeared to have promise as a therapeutic agent for the treatment of anxiety disorders such as autism spectrum disorders (139). However, its failure in preclinical development led to the abandonment of the nonpeptide OT agonist programme at Pfizer (44). WAY 267464 is now available from TOCRIS. Presently, there are no other companies pursuing the development of nonpeptide OT agonists.

## The use of radiolabelled molecules, agonists and antagonists for characterising receptor affinities for OT and VP (Tables 11 and 12)

**Table 11:** Common Agonists to Oxytocin (OT)/Arginine Vasopressin (AVP) Receptors^a^.

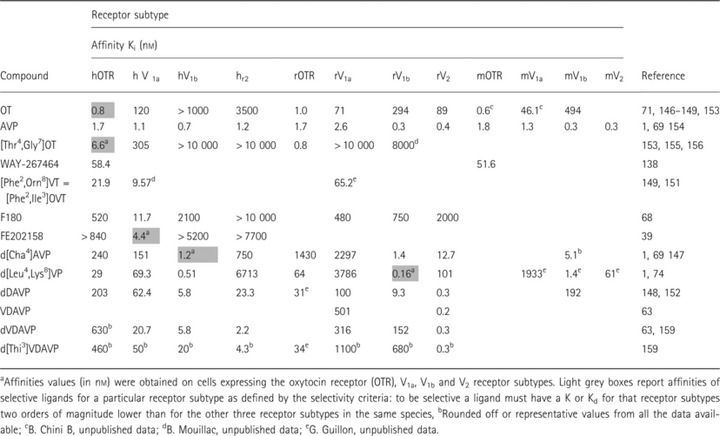

**Table 12:** Common Antagonists to Oxytocin (OT)/Arginine Vasopressin (AVP) Receptors^a^.

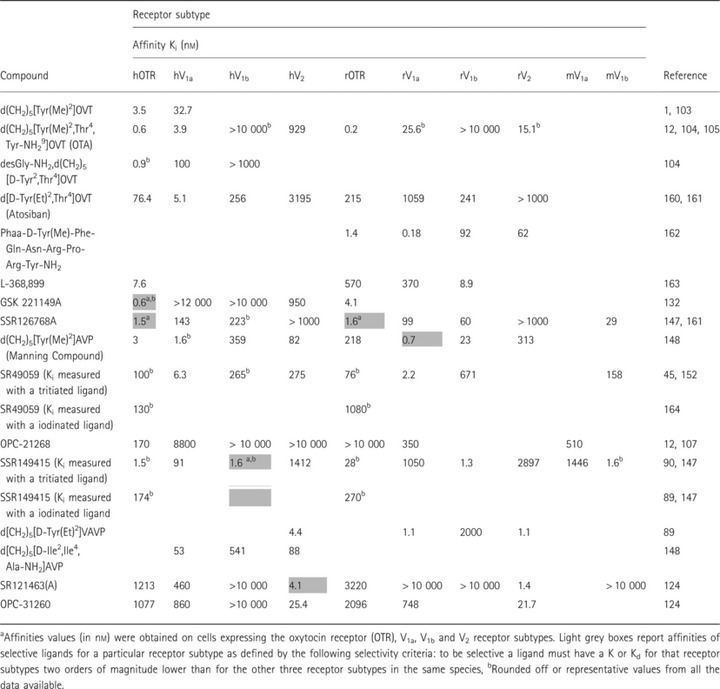

### Some history

Initially in the 1960, the affinity, selectivity and potency of analogues for the different VP/OT receptors were deduced by *in vivo* bioassays such as oxytocic, antidiuretic and pressor tests (see above), which reflected their activity through the OT receptor, V_2_ and V_1a_ receptors, respectively. The characterisations performed at that time did not take into account the V_1b_ receptor, which was discovered only in the 1980s (142). As noted above, this led to a long delay in the discovery of selective ligands for the V_1b_ receptor. Nevertheless, the use of bioassays allowed the identification of key structure/function relationships of a large number of analogues and still represents a milestone in our understanding of OT/AVP selectivity (1, 7–18). Furthermore, because the data obtained using these *in vivo* tests integrated several ADME (Absorption, Distribution, Metabolism and Elimination) parameters, the values obtained reflect the *in vivo* physiological activity of the peptide being studied. These values sometimes differed from those obtained by *in vitro* pharmacological tests.

In the 1970s, the development of radiolabelled VP/OT analogues (143) and the discovery of second messenger cascades such as cAMP (67), calcium and inositol phosphate (144) made possible the determination of more reliable pharmacological parameters reflecting more precisely the interaction between analogues and their specific receptors. Binding assays with radiolabelled ligands conducted on plasma membrane preparations allowed the determination of the affinity (K_d_) of a given molecule for a given VP/OT receptor subtype, a parameter which intrinsiquely characterises the analogue/receptor association (145). Second messenger measurements allowed the characterisation of its functional activity in order to measure precise functional effects. Comparison of the affinities of one analogue for the all receptors of the VP/OT family allowed the determination of its selectivity towards a given receptor isoform. Moreover, the ability of a given analogue to activate, inhibit or leave unaffected second messenger production in cell cultures, provided important insights into its pharmacological status (agonist, partial agonist, pure antagonist, inverse agonist). Such classical pharmacological assays have efficiently served the scientific community for the last three decades and allowed the characterisation of the numerous VP/OT analogues designed and synthesised during this period (1).

## Conundrums posed by pharmacological data

From all the *in vitro* and *in vivo* pharmacological studies carried out on OT and VP agonists and antagonists, three intriguing features have emerged; namely: (i) lack of receptor selectivity; (ii) species differences; and (iii) *in vitro in vivo* difference. In Tables 11 and 12, we have listed the most commonly used agonists and antagonists available for each VP/OT receptor isoform in three mammalian species: human, rat and mouse.

The affinities (K_i_) of these isoforms have been measured using classical pharmacological tests and their agonist or antagonists properties determined using classical second messenger assays. As proposed in previous reviews (156, 157), receptor subtype selectivity can be defined, within a single species, on the basis of the ability of a compound to bind to a single VP/OT receptor isoform with a nanomolar affinity, at the same time displaying, for the three other receptor isoforms, an affinity at least two orders of magnitude lower. The compounds that fullfill these requirements are highlighted with light grey in Tables 11 and 12. It immediately appears from this criterion that only a very few analogues are selective. A major problem is also that selectivity is not conserved among species as a result of subtle but nevertheless crucial differences in receptor pharmacology. Despite these limitations, the use of selective compounds still represents the best experimental strategy to unambiguously characterise VP/OT receptors in a given biological sample, keeping in mind that receptor selectivity for any given compound is: (i) strictly dependent upon the receptor species considered; (ii) usually lost if high doses (100-fold the K_i_) of a selective compound are used; and (iii) dependent upon the biological models tested. Experiments performed on membrane preparations or on cell cultures generally need lower concentrations of selective analogue compared to experiments performed on organ slices, where drugs need to diffuse within the tissues and may be rapidly degraded.

It should also be noted that the pharmacological profile of any given compound determined by classical tests on membranes or cell models cannot be directly translated *in vivo* without adequate controls. Adsorption, distribution in different biological compartments, and metabolism greatly interfere with the biological activity of drugs, sometimes completely altering their pharmacological properties.

For example, [Phe^2^Orn^8^]VT (also known as [Phe^2^]OVT), which does not display any V_1a_ selectivity in classical binding experiments (Table 11), has been characterised as a selective V_1a_ agonist *in vivo* in rats (Table 4) (81).

Concerning selective agonists, it should also be noted that a major difference exists between the two natural hormones, OT and VP. Although OT is selective for the human OT receptor, VP is not, because it binds with similar affinities to V_1a_, V_1b_ V_2_ and OT receptors. This may explain why VP may trigger physiological functions *in vivo* via OT receptors, as described previously (158). However, fully characterised selective agonists for human V_1a_ receptor (F 180, FE202158), human V_1b_ receptor (d[Cha^4^]AVP); rat V_1b_ receptor (d[Leu^4^,Lys^8^]AVP), rat OT receptor [Thr^4^,Gly^7^]OT and rat V_2_ receptor (dDAVP) are now available (Table 11). For the rat, V_2_ receptor d[Thi^3^]VDAVP (15, 159) appears to be the best selective agonist as a result of its good V_2_ versus V_1b_ selectivity.

Among the several antagonists reported and currently employed, only a few have been fully characterised and have been demonstrated to be selective whithin a species (Table 12).

Among the OT receptor antagonists, SSR126768A has been shown to be a very selective antagonist for both human and rat OTR and GSK 221149A for human OT receptor. Manning compound is relatively selective for the rat V_1a_ receptor (but not for the human V_1a_ receptor) for which SSR49059 should be preferred. Finally, SSR149415 (147) is selective for both the human and the rat V_1b_ receptor isoforms, whereas SSR121463(A) is highly selective for the human V_2_ receptor. Concerning the SSR149415 and SSR49059, it should be noted that different laboratories have obtained different values for their affinities, probably depending on the binding assay employed (i.e. competition against a radioactive agonist or antagonist) (89). In our opinion, the values obtained in competition experiments using radiolabelled agonists will better correlate with biological antagonistic activity *in vitro* and *in vivo* and should be preferred.

Until now, other analogues commonly employed as ‘selective’ have not been fully characterised and, when they are used at high doses, could lead to ambiguous results in species in which their pharmacological properties have not been assessed. It should be noted that the pharmacology of OT/VP analogues on mouse receptors is still very limited, representing a gap that needs to be filled; in particular, for the relevance that genetically-modified mouse models have acquired in translational medicine.

The lack of selective analogues for some rat, mouse and human receptor isoforms makes the design and synthesis of new molecules very necessary. The restriction of radioactivity approaches in laboratory practice and the need to easily test a large number of molecules led to the development of new assays using the gene reporters. Such tests using the measurement of reporter gene activities allows an easy screening of a large number of molecules and rapid identification of ‘lead molecules’ (83).

Yet, these ‘*in vitro* methodologies’ also have some limitations. First, they can be used only in transfected cells and not in native models. Moreover, according to the second messenger cascade associated with the receptor being considered (cAMP for the V_2_, InsP3 for the V_1a_, V_1b_ and OT receptor isoforms), such assays require the use of different reporter genes. This may introduce a bias in the determination of receptor selectively. It is also well known that assays using luciferase gene expression and luciferase activity measurements involve a strong amplification of the initial receptor-mediated second messenger accumulation. This prevents the good determination of the agonist or partial agonist properties of the analogue tested. One needs to be aware of the limitations of these recent ‘*in vitro* methodologies’ and to verify, using classical pharmacological tests, the selectivity, affinity and functional potencies of the lead compounds characterised by this approach. Obviously, to move to clinical development, the best approach would be to test the compounds of interest by *in vivo* technologies similar to those used to evaluate virtually all of the peptides in Tables 1, 3–8.

## New technologies for screening more selective VP/OT analogues

Recently, new physical techniques involving label-free biosensors have been proposed for pharmacological screening of muscarinic and corticotrophic analogues (165). These methods are based on the measurement of cell shape changes induced by ligand-receptor interactions. Such techniques have the advantage of being performed on native cells and do not require the use of radioactive molecules. Their efficiency for testing new VP/OT molecules may represent another alternative for screening new analogues.

Finally, a new bioluminescence or fluorescence resonance energy transfer (BRET or FRET) approach in which analogues could be screened for their capability to promote receptor coupling and activation of single G-protein isoforms has been recently applied to the human OT receptor (166). This technique allows the precise characterisation of which G protein is associated with which receptor isoform. Thus, for example, d(CH_2_)_5_[D-2-Nal^2^,Thr^4^,Tyr-NH_2_^9^]OVT (OTA) and atosiban (160) (Table 12) were found to be entirely biased respectively toward Gi1 or Gi3 activation (166). However, this technique cannot be used on native tissues or primary cultures.

## The recent development of fluorescent ligands for a better knowledge of central and peripheral VP/OT receptors

### Design and use of classical fluorophores

Receptors of the AVP and OT family are important in the regulation of the stress processes (167). Centrally, the V_1a_, V_1b_ and OT receptors have been involved in stress and especially in learning and memory processes. Important data have been obtained by the use of knockout animals but, after a period of cloning and pharmacological characterisation in the last decade, it became necessary to elucidate the distribution of these receptors to better understand their central functions *in vivo*.

Although several publications describe the AVP V_1a_, V_2_ and OT receptor distribution by using autoradiography (105, 168, 169) or immunodetection (170), the lack of selective V_1b_ radio-labelled VP analogues or of receptor antibodies has hindered progress in the detection of receptor distribution in native tissues. Results obtained by molecular approaches such as reverse transcriptase-polymerase chain reaction (62, 88) or mRNA detection by *in situ* hybridisation (171–173), although more accurate, did not provide clear information regarding the brain regions detected by immunostaining. Thus, developing fluorescent ligands to decipher AVP receptor distribution in the brain and at the periphery, and to study molecular interactions such as receptor dimerisation, appeared as an absolute necessity.

Various fluorescent analogues of AVP and OT have been synthesised for several receptors of the VP/OT family (174). Thus, good fluorescent V_1a_ and OT ligands have been produced, although no good fluorescent specific ligand was available to selectively detect central and peripheral V_1b_ receptors.

In our previous work (69, 73, 74), by replacing the glutamine^4^ of the natural AVP with a cyclohexylalanine or a leucine, the arginine^8^ by a lysine and by removing the NH_2_ of the cysteine^1^ to increase stability towards aminopeptidases, we produced analogues showing an increased selectivity for the V_1b_ receptors (Table 5). d[Leu^4^,Lys^8^]VP (Peptide **4**; Table 5) was found to be selective for the rat V_1b_ receptors and, to a lesser extent, for the human hV_1b_ receptors, conserving a nanomolar affinity for these receptor isoforms (73, 74). We have taken advantage of the Lys^8^ residue in d[Leu^4^,Lys^8^]VP with its epsilon NH_2_ group to introduce fluorophores on its side chain. This allowed us to create fluorescent tools that would conserve the pharmacology of the d[Leu^4^,Lys^8^]VP, to resist degradation and to selectively decorate the plasma membrane of Chinese hamster ovary cells expressing V_1b_ and/or OT receptors with an excellent resolution (47).

Different fluorophores were attached to the d[Leu^4^,Lys^8^]VP: First, the antraniloyl group (Atn), a small fluorescent molecule of 97 Da highly sensitive to microenvironmental changes (175) may also be a good donor in FRET experiments to identify V_1b_ receptor homodimers *in vivo*. We have also selected the Alexas (Molecular Probes) for their brightness and their resistance to photobleaching (176). We have used Alexa 488 and Alexa 647, with the latter being one of the brightest fluorescent molecules reported so far (177).

The pharmacological properties (binding, coupling to phospholipase C) of fluorescent analogues of d[Leu^4^,Lys^8^]VP indicate that they conserved a very good selectivity for V_1b_ versus V_1a_ and V_2_ receptors, and remained full agonists. These properties allow receptor labelling and measurement of biological activity at the cellular level. Thus, these new fluorescent analogues are promising tools for the detection of functional V_1b_ or OT receptors in human (47) and in rat native tissues.

### Use of long life fluorophores

However, it should be noted that, except for very recent ones, all the ligands previously reported were designed with classical fluorophores, exhibiting short-lived fluorescence properties (fluorescence half-time life in the 10 ns range). Most of them were essentially used to follow internalisation in cell lines (146, 178) or to label receptors in a native context (179). Interestingly, a first nonpeptide antagonist with a nanomolar affinity for the human V_2_ receptor has been developed (180). This ligand will find application in fluorescence polarisation-based binding assays aiming to screen for small organic molecule libraries.

Recently, fluorescence-based strategies have been extensively used to study molecular interactions. Thus, the FRET approach was used to demonstrate G protein-coupled receptors oligomerisation (181, 182). Regarding AVP and OT receptors, various experimental approaches based on chimeric receptor expression in cell lines have been developed to analyse receptor oligomers. The studies have used receptors fused either to small tags recognised by fluorescent antibodies (183, 184), or to bioluminescent or fluorescent proteins (185, 186), or to suicide enzymes (48). However, these strategies were not relevant for proving the existence of such receptor complexes in native tissues. Therefore, a FRET strategy based on the indirect labelling of receptors with fluorescent donor and acceptor ligands has been recently developed (48). Unfortunately, because of the overlap between excitation and emission spectra of the donor and acceptor fluorophores on one hand, and of the high autofluorescence of the biological preparation on the other hand, specific FRET could hardly be detected. To improve the signal-to-noise ratio, lanthanide cryptate-labelled ligands were designed and characterised. Despite the size of the cage, these ligands still display very good affinities for the V_1a_ and OT receptors (48). Lanthanide cryptates display interesting fluorescent properties because they have a fluorescent half-time life of approximately 1 ms (i.e. 100 000-fold greater than classical fluorophores), allowing time-resolved FRET experiments to be set up (187). Experiments using these new probes have been performed not only on AVP V_1a_ and V_2_ receptors and on OT receptors expressed in cell lines, but also on OT receptors naturally expressed in lactating rat mammary gland. The sensitivity is such that it has been possible to prove the existence of OT receptor dimers in this latter native tissue (48).

These newly-synthesised ligands and those that exhibit high quantum yield have also been used to develop original binding assays. These assays, based on time-resolved FRET between compatible fluorophores carried by tagged receptors and ligands, display very good sensitivities and are safer than radioactive-based assays (188–190).

## Therapeutics uses of peptide and nonpeptide oxytocin and vasopressin agonists and antagonists (Table 13)

**13:** Food and Drug Administration Approved Peptide and Nonpeptide Drugs from the Oxytocin (OT)/Arginine Vasopressin (AVP) Field.

OT/AVP field	
Peptides^a^	Nonpeptides^b^
Carbetocin (Duratocin, Depotocin, Sofla, Pabal)	Conivaptan hydrochloride (Vaprisol)
Desmopressin (Minirin, DDAVP)	Tolvaptan
Ornithine vasopressin (Ornipressin, POR-8)	
Lypressin (Diapid, LVP)	
Oxytocin	
Terlipressin (glypressin)	
Vasopressin (Pitressin)	
Atosiban (in Europe)	

a See Reichert (194). ^b^See Ferguson-Myrthil (115).

Table 13 lists the seven peptides and two nonpeptide drugs in the OT and AVP field that have been approved for therapeutic use. Numerous recent studies point to the use of OT as a potential new therapy for the treatment of a broad range of psychiatric disorders (24, 33, 34). Also, a very recent report suggests the exciting prospect that OT may have potential for the treatment of human obesity and type 2 diabetes (191).

To date, only two nonpeptides, the VP V_2_/V_1a_ antagonist conivaptan and the VP V_2_ antagonist tolvaptan have been approved for clinical use (112–115, 123, 192). The nonpeptide OT antagonist retosiban (132, 133) is currently in a Phase II clinical trial. Clinical trials with other nonpeptide VP, V_1a_ and V_1b_ antagonists shown in Table 9 and with the other OT antagonists and the OT agonist shown in Table 10 have been terminated (44). No new nonpeptides in this field are currently in clinical trial. Thus, the early promise of nonpeptides as therapeutic agents in this field (45, 46) has clearly not been realised. This should be a cautionary tale for those in pharmaceutical companies (138), in granting agencies (139) and on study sections (44) who have strongly promoted the development of nonpeptides over peptides as therapeutic agents. This philosophy led to the abandonment of peptides as potential drugs by Big Pharma almost two decades ago. Clearly, it is now time for Big Pharma to reassess the value of peptides as therapeutic drugs (44). In this regard, recent progress in the development of peptide drugs (193, 194) provides very compelling support for the thesis that peptides are clearly superior to nonpeptides as therapeutic agents, thus bolstering the case for continued support for the design and synthesis of peptides as potential therapies. In this regard, in the OT field, there is an urgent need for functionally selective OT ligands (166) and for a long lasting OT agonist as a potential therapy for the treatment of autism and other anxiety disorders (139).

## Research uses of peptides

During the period 1980–2012, over 3000 samples of OT and AVP agonists and antagonists from the Manning laboratory have been and continue to be donated as research tools to over 700 investigators (some multiple times) in the USA and worldwide for their own independent studies. Studies carried out with these donated peptides and with those purchased from commercial suppliers, such as Sigma, Bachem and Peninsula, have resulted in more than 2000 publications by these and other investigators during this period. Examples of studies carried out since 2008 with some of these peptides are available (90, 117, 195–246).

## Research uses of nonpeptides

With the exception of the Sanofi nonpeptide V_2_ antagonist satavaptan, all of the nonpeptide AVP antagonists listed in Table 9 are now available from Tocris or other suppliers. To date, a small number of studies that have utilised the Sanofi V_1a_ antagonist relcovaptan, the Sanofi V_1b_ antagonist nelivaptan and the Sanofi V_2_ antagonist satavaptan have been reported (117–120, 125, 126) (Table 9).

The two Merck nonpeptide OT antagonists L-368,899 and L-371,257 and the Pfizer nonpeptide OT agonist Way-267464 shown in Table 10 are all available from Tocris. The Glaxo SmithKline nonpeptide OT antagonist GSK-2211149A (Retosiban) is also available from a number of suppliers. The Pfizer nonpeptide OT antagonist WAY-162720 is not yet commercially available. A number of studies that have utilised some of these nonpeptide ligands as research tools have been reported (138–141, 145) (Table 10).

## Conclusions

In our 2008 review (1), we examined the status (as of 2007) of both peptide and nonpeptide agonists and antagonists of the OT receptor and of the VP V_1a_, V_1b_ and V_2_ receptors as: (i) research tools and (ii) therapeutic agents. Although the research uses of both peptide and nonpeptide ligands have continued to grow during the intervening 4 years, by contrast, the therapeutic development of nonpeptide AVP and OT antagonists has been drastically curtailed. Merck, Pfizer and Sanofi have all abandoned their nonpeptide programmes. The nonpeptide V_2_/V_1a_ the antagonist, conivaptan and the nonpeptide V_2_ antagonist tolvaptan, which have been approved by the Food and Drug Administration, have not as yet found widespread acceptance in the clinic (113, 192). Pfizer has also abandoned its nonpeptide OT agonist programme (44). It remains to be seen how the Glaxo SmithKline nonpeptide OT antagonist retosiban will fare in its current Phase II clinical trial. All in all, since our 2008 review (1), interest in the development of nonpeptides as therapeutics has greatly diminished. On the other hand, as noted above, Ferring has a promising V_1a_ agonist (Table 4) and a promising OT agonist (Table 1), awaiting clinical development. The design and synthesis of: (i) functionally selective OT peptides and (ii) of a long lasting OT analog as a potential therapy for autism spectrum disorders and other anxiety disorders remain as pressing needs in this field. Both OT and VP peptides and nonpeptides are continuing to be very valuable research tools. In this regard, we have addressed here the issues of the: (i) lack of receptor selectivity, (ii) species differences and (iii) *in vitro–in vivo* differences, all of which need to be taken into account when using a given peptide or nonpeptide ligand as a research tool. Finally, the development of new fluorescent ligands as powerful new tools for localising and characterising OT and VP receptors, which we have presented here, points to the continued usefulness of OT and AVP peptide ligands (agonists, antagonists and fluorescent derivatives) as incisive molecular pharmacological probes.
